# Tomasz Drobnik (1858–1901)—An Assistant Who Equalled His Masters

**DOI:** 10.1007/s00268-013-2351-8

**Published:** 2013-12-05

**Authors:** Krzysztof Pietrzak, Andrzej Grzybowski, Jacek Kaczmarczyk

**Affiliations:** 1 Department of Orthopaedics and Traumatology, University of Medical Sciences, 28 Czerwca 1956 135/147, 61-545 Poznan, Poland; 2 Department of Ophthalmology, Poznań City Hospital, Poznan, Poland; 3 Department of Ophthalmology, University of Warmia and Mazury, Olsztyn, Poland

## Introduction

Tomasz Drobnik (1858–1901) was a Polish-born practicing surgeon who developed new surgical techniques despite working under adverse political conditions. Having completed his medical studies in Würzburg, he worked in a few German hospitals, among others in Strasbourg, where he developed new principles of thyroid surgery, particularly a new method of accessing the gland. Beginning in 1887 Drobnik worked in Köningsberg, in Professor Mikulicz-Radecki’s hospital, where he developed a new method of surgical repair of cleft palate, as well as a technique of facial nerve crossover anastomosis, probably the first in the world. He continued his research and practice in Poznań, where he moved in 1890. This is where he laid the foundations of a new branch of medicine, today known as neuro-orthopedics. He was the first to perform a successful tendon transfer, subperiosteal implantation of a tendon, and vascularized muscle transfer. His perception of musculoskeletal system disorders as being dynamic led him to develop a cutting-edge method of club foot treatment. Drobnik achieved all this on his own while working as an assistant in a Poznań Town Hospital devoid of the research facilities of academic medical centers. Alongside his research, he was also engaged in community service. He was equally devoted to his Polish and German patients, even at the time of escalation of national animosities.

## Early years and education

**Fig. 1:**
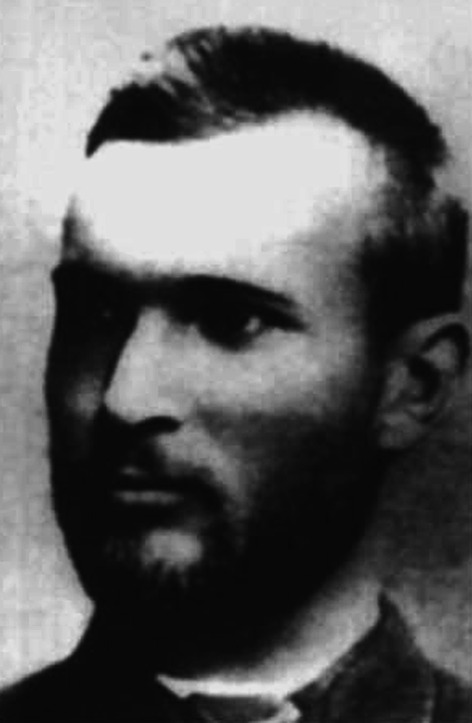
Tomasz Drobnik (1858–1901)

Tomasz Drobnik (Fig. 1) was born on 6 September 1858 in Pleszewo, a once Polish territory then under Prussian rule. His parents were Jan, a land owner, and Emilia, née Kulesiewicz, both of the middle class. Drobnik began his education in Polish Royal Gymnasium in Ostrów. Later he moved to Poznań, where he graduated from St Mary Magdalene Gymnasium. In 1880 he began medical studies in Wrocław. He was one of very few Poles who were allowed to be voluntary assistants in the Department of Anatomy. He continued his studies in Würzburg [1], where he was awarded a degree as Doctor of Medicine [2].

Having completed his military service, he worked in Wrocław for two years in the Department of Anatomy and then moved to Strasbourg to work with J. G Jossel, who taught surgical anatomy. There he wrote three articles on the anatomy of the thyroid. In the first of these, based on 50 anatomical specimens, he described the normal course of the inferior thyroid artery and the recurrent laryngeal nerve [3], showing that the they may vary considerably. Drobnik’s descriptions proved to be more precise than those of Theodor Billroth and Franz Köning [4], then considered standard. Afterwards, Drobnik suggested a new method of accessing the inferior thyroid artery, between the sternal head and the clavicle head of the sternocleidomastoid muscle [5]. As this method was both simple and required no assistance, it soon gained recognition as better than Langenbeck–Wölfler’s access [4], widely used at that time. In the same article he postulated that the inferior thyroid artery should be ligated between the ascending cervical artery and the superficial cervical artery in order to avoid damage to the branches of the sympathetic nerves. Drobnik’s suggestions were followed by Jan Mikulicz-Radecki to perform ligation of thyroid arteries to treat Graves’ disease and then by Friedrich Trendelenburg and Emil Theodor Kocher [1, 6].

In his next publication [7] Drobnik described the position and course of the sympathetic trunk branches around the inferior thyroid artery. He proved the existence of many connections between the branches of the vagus nerve and the sympathetic trunk, especially in the area of the thyroid.

His publications were widely acknowledged. In the years 1887–1890 Drobnik worked in the Department of Clinical Surgery at the Univeristy of Köningsberg, where he started his surgical training under the guidance of professor J. Mikulicz-Radecki. On the basis of specimens, he extensively researched a new method for treating facial nerve neuralgia proposed by Mikulicz. Additionally, he proved that clinical symptoms of both inferior thyroid artery ligation and thyroidectomy were not identical to symptoms of myxedema. While in Köningsberg, Drobnik rarely performed surgeries on his own but rather focused on assisting.

In general opinion, Drobnik is credited with performing the first facial nerve crossover anastomosis [8]. He is believed to have performed the surgery in 1879, at the age of 21 [9]. This is clearly improbable, as at that time Drobnik had not even started his medical training. However, problems with establishing the exact date do not undermine the fact that Drobnik did perform this surgery, probably as the first in history. Drobnik reported clinical improvement after the surgery.

While in Köningsberg, he had extensively studied the facial nerve, and the surgery may have taken place between 1887 and 1890. At the same time he studied and theoretically developed other surgical treatments, for example cleft lip repair, which he published a few years later [10]. Mikulicz allowed him to perform this surgery only once. The situation might have been the same with facial nerve surgery. The problems with determining the exact date make it impossible to unequivocally settle the question of Drobnik’s primacy in this case. In 1894 Charles Ballance made an unsuccessful attempt at the reconstruction of the facial nerve by shortening its course [8]. Nonetheless, Drobnik remains, beyond dispute, one of the world pioneers of facial nerve surgery.

## In Poznań

**Fig. 2:**
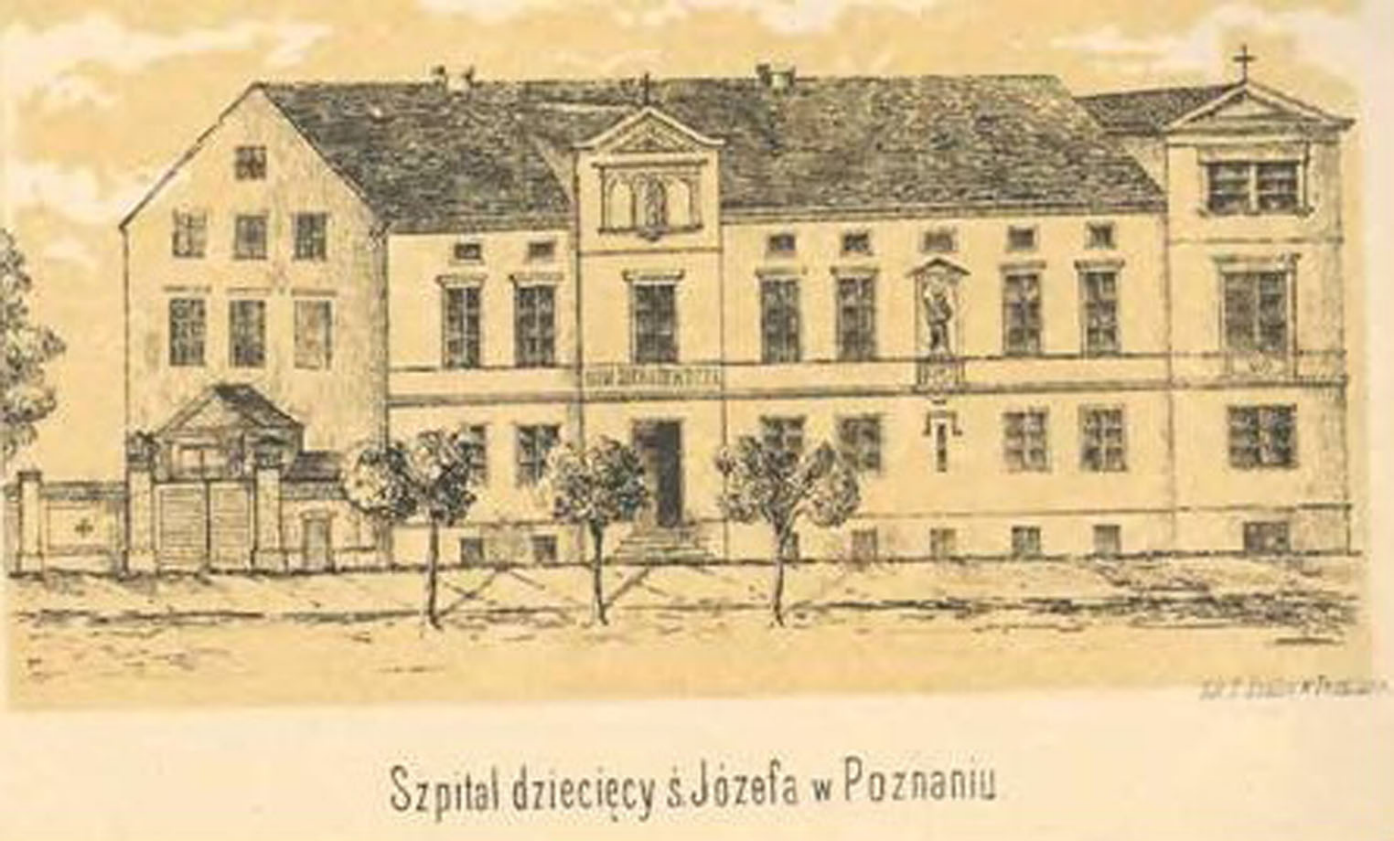
St Joseph Hospital in Poznań

Drobnik returned to Poznań in 1890 to hold the chair of the Department of Pediatric Surgery in St Joseph’s Hospital (Fig. 2). The conditions in the hospital were extremely harsh. There were only 12 beds, and only one was for surgical patients. Neither sterilization nor a separate operating room existed. Drobnik had to perform surgeries in the same room where outpatients were treated, often for pus infections. It is all the more astonishing that in such conditions Drobnik’s results came close to those achieved in the largest German centers. This was due both to strict observance of aseptic technique and perfect organization. There was no assistant, and anaesthesia was given by a nun. With time, the department grew to 22 beds, and the first assistant, a gynecologist, came on the scene. This allowed Drobnik to broaden the scope of operations to include abdominal surgery. He performed hundreds of surgeries, which soon earned him the name of the first surgeon of Poznań. In his practice he employed cutting-edge techniques, which he quite often developed and enhanced. The only thing he truly regretted was lack of research assistance to carry out theoretical studies in the field of surgery [11].

Drobnik proposed an entirely novel method of surgical repair of cleft palate [10]. He pointed out that the reconstruction should include not only the skin but also the muscles of the upper lip. Consequently, he separated the upper lip from the palate with a large incision. This approach to cleft lip repair came to be widely used several dozen years after Drobnik. He also advocated the use of tracheotomy in diphtheria and employed it successfully in 176 cases [12].

Drobnik also proposed that reducible hernias in children should be treated by removing the hernia sac at the place of the protrusion and then closing the incision with sutures [13]. This was a departure from the then standard practice of excising the whole hernia sac. He used this technique in 55 young patients, and there were no cases of recurrence [13]. Drobnik was also well aware of the importance of fluid replacement in patients undergoing abdominal surgery [14]. His knowledge of fluid replacement and prudence in fluid management resembled today’s standards and surpassed the best medical practices of his time. He was a frim advocate of fast surgery in cases of gastrointestinal perforations [15]. He estimated that to save such patients surgery should be carried out within 10 h of the perforation. However, the dominant option at that time was conservative treatment [4].

## Work in orthopedic surgery

Drobnik was genuinely interested in orthopedics. At that time there was no Department of Orthopaedics in Poznań, the first one was established in 1913. In 1890 Drobnik published an article on the treatment of club foot [16], in which he reported 6 cases. He pointed out that the condition involved muscle disorders, particularly of the tibalis posterior and flexor hallucis longus. During operations Drobnik lengthened these muscles. He did not suture the tendons because he wanted scars to appear. Also, contrary to existing practice, he did not apply casts to the legs. Instead, he used two elastic bandages, which allowed gradual correction of the foot position on a daily basis. The correction was reinforced by changing the position of the bandages, which was a very original idea at that time. A similar approach is nowadays used in the Ponseti method, where the deformity is redressed by applying a series of casts. Drobnik did not perform Achilles’ tendon lengthening in children, only in teenagers and adults. In his research on club foot he was the first to discover that claw toes result from the flexor hallucis longus muscle being hypertonic. His approach to thinking about foot deformities as a result of muscle imbalance was innovative, as was his idea of gradual correction of the foot shape. Before that, club foot was perceived as a static condition and was treated with a one-stage procedure.

Drobnik’s perception of illness as a result of dynamic processes gave rise to a series of publications on surgical treatment of palsies. Drobnik was the first in the world to perform a successful tendon transfer. The patient was a seven-year-old girl suffering from postpoliomyelitis paralysis. Drobnik transposed the tendon of the extensor hallucis longus to the extensor digitorum longus. The result was presented at the session of the Medical Section of the Poznań Society of the Fiends of Learning on 4 December 1892. A year earlier an attempt at tendon transfer had been made by Carl Nicoladoni (1847–1902), but it failed [17].

Drobnik presented further cases at subsequent sessions of the Friends of Learning. He successfully treated paralytic pes calcaneus by transposing the tendon of the flexor digitorum longus and fibularis longus to the heel bone. However, his attempt at replacing the tibalis posterior muscle with extensor hallucis longus muscle failed, because of the tendon shape.

In 1894, Drobnik again came to the forefront of orthopedic surgery, performing the first implantation of a transplanted tendon into bone. He transferred the tibalis anterior to the cuboid bone.

Undoubtedly, Drobnik was the first to perform tendon transfer in the upper limb. The surgery was performed on a 4.5-year-old girl with postpoliomyelitis paralysis of the radial nerve. He transferred the extensor carpi radialis brevis to the extensor pollicis longus and extensor carpi radialis longus to the extensor digitorum [18]. The girl regained the ability to grasp things. Drobnik’s surgery defied the then widespread belief that radial nerve palsy cannot be treated. In 1894 Drobnik, again as the first in history, performed vascularized muscle transfer [19]. During the patients’ rehabilitation he also noticed that straining the treated limb had considerable impact on improving its condition, temperature, and blood supply. He even pointed out that proprioception can be restored by treading the floor with a bare foot [18]. It needs to be highlighted that Drobnik made all these accomplishments on his own, without support of a clinical department or a research team.

Next, Drobnik devised a method for the treatment of rickets-induced bow-legs. At that time the treatment included breaking the shins with a special apparatus, which resulted in severely traumatized soft tissue. The gastrocnemius muscle was often damaged during the procedure. Drobnik, on the other hand, performed corrective osteotomy of shins and lengthening of the Achilles’ tendon. He also corrected tibia that were curved backwards [20]. This method prevented complications, muscle atrophy, and wide scarring, which often thwarted doctors’ efforts.

Drobnik extensively studied bone tuberculosis. At that time Europe was full of high hopes that Koch’s discovery of tuberculin would eradicate tuberculosis once and for all. Drobnik was very skeptical about it, which later proved right. His surgical treatment of bone tuberculosis was based on the observation that large changes in the bones were quite often coupled with good general well-being. Therefore, he was aware that tuberculosis virulence varied. He operated very conservatively, trying to protect the healthy tissue around the changes. In the early stages of synovial tuberculosis of the knee he removed only the synovial membrane. Additionally, he widely applied punctures, drains, and skin transplants to compensate for removed tissue [17, 21].

Drobnik was also interested in wound treatment. He advocated skin incisions parallel to the elastic fibers. He was a pioneer of modern treatment of gunshot wounds and soiled wounds. He tried to make them similar to surgical wounds by wide excision of the edges, drainage, and immobilization [22]. He claimed that when the edge cannot be excised, it should be cut open with a wide incision and drained to facilitate outflow of exudate. This approach was not followed until the First World War and became widely used only at the outset of the Second World War.

In 1899 Drobnik won a competition to chair the Surgery Department in Poznań City Hospital, where he worked until 1901. This is all the more remarkable as the appointment required approval of the Poznań City Council, which at that time was dominated by strongly anti-Polish Germans. However, even they could not deny Drobnik’s position as the first surgeon of Poznań. Drobnik had only one assistant, a German. After initial distrust, their relationship began to flourish. Drobnik still operated in his old ward in St Joseph Hospital. Additionally, from 1895 he ran his own outpatient clinic at the Old Market Square in Poznań.

Drobnik dedicated himself to community service. He was deeply engaged not only in activities of medical societies but also in patriotic activities, fighting for autonomy and self-determination of Poles living in the eastern provinces of Germany. As part of his community service, he organized summer trips for children. He also published in the popular press. All of this earned him wide popularity and recognition, even among Germans, despite the fact that they did not approve of Drobnik’s patriotism. Even at the height of the anti-Polish campaign, they could not but admire his surgical achievements. So high was their regard that the German authorities considered giving Drobnik an academic position to enable him to obtain further medical degrees [6].

Hard work in several institutions and engagement in community service imposed tremendous strain on Drobnik’s health. For the last two years of his life he suffered from chest pains, which he kept secret. Still, he would not rest. He died of a heart attack on 22 May 1901. He was buried in Poznań, and his funeral turned into a great manifestation of Polish patriotism but in fact all nations were united in mourning. His funeral procession was led by Poznań’s German mayor [23].

Drobnik was married to Helena, née Szuman, from a gentry family. They had three sons. Drobnik immensely enjoyed resting in his house in Kobylnica near Poznań, where he could revel in nature [24].

## Conclusions

Drobnik’s professional career focused on practical surgery. For many years he was either an assistant or the only surgeon of the ward. The position of chief surgeon, for which he was perfectly suited and which was just a prelude to his further career, was granted to him only for the last two years of his life. Most of the surgical treatments he developed were based on his individual work. Despite these difficulties, coupled with the need to provide for his family, and engagement in community and patriotic duties, he earned an important place in the history of surgery and its youngest child—orthopedics.
